# Kinetic and homology model analysis of diaminopimelate decarboxylase from *Cyanothece* sp. ATCC 51142: unveiling a key enzyme in lysine biosynthesis

**DOI:** 10.1042/BSR20253430

**Published:** 2025-09-18

**Authors:** Zhi-Min Li, Suhang Chen, Weikang Luo, Fang Wang, Siqi Wang, Liyang Huang, Xinyue Xiong, Congcong Xie, Zhimin Li

**Affiliations:** 1 College of Chemistry and Materials, Jiangxi Agricultural University, Nanchang, Jiangxi, 330045, China; 2 College of Bioscience and Bioengineering, Jiangxi Provincial Key Laboratory for Postharvest Storage and Preservation of Fruits and Vegetables, Jiangxi Agricultural University, Nanchang, Jiangxi, 330045, China; 3 Jiangxi Provincial Psychiatric Hospital, Nanchang, Jiangxi, 330000, China; 4 School of Biological and Environmental Engineering, Jingdezhen University, Jingdezhen, Jiangxi, 333000, China

**Keywords:** *Cyanothece *sp. ATCC 51142, diaminopimelate decarboxylase, enzymatic kinetics, homology modeling, lysine biosynthesis, molecular docking

## Abstract

Diaminopimelate decarboxylase (DAPDC), a pyridoxal 5′-phosphate (PLP)-dependent enzyme, catalyzes the decarboxylation of diaminopimelate (DAP) to yield L-lysine, a key step in lysine biosynthesis. This present study presents a preliminary characterization of DAPDC encoded by the *cce1351* gene in *Cyanothece* sp. ATCC 51142 (CsDAPDC), focusing on its biochemical properties and model structure characteristics. The enzyme exhibited a peak activity at 30°C and pH 8.0, and the catalytic constant (*k*_cat_) and substrate binding affinity Michaelis constant (*K*_M_) were determined as 1.68 s^-1^ and 1.20 mM at the above-mentioned condition, respectively. Homology modeling and molecular docking analysis revealed that Gly286, Gly330, Tyr428, and Asp118 interacted with the PLP cofactor, and Ser249, Tyr372, and Tyr428 interacted with the DAP substrate. Additionally, Cys399, Glu400, and Tyr436 from the other monomer were also involved in binding DAP and PLP. Site-directed mutagenesis confirmed the functional roles of these key residues in catalysis. This work provides valuable insights into the catalytic mechanism of CsDAPDC and highlights the enzyme’s potential for applications in metabolic engineering of cyanobacteria for enhanced lysine production.

## Introduction

*Cyanothece* sp. ATCC 51142, a filamentous nitrogen-fixing cyanobacterium, serves as a model organism for studying metabolic pathways due to its unique capacity to simultaneously conduct oxygenic photosynthesis and nitrogen fixation [1–3]. This strain has been extensively studied for its physiological and metabolic adaptability, allowing it to thrive in various environmental conditions [4]. Recently, *Cyanothece* sp. ATCC 51142 has emerged as a candidate for biotechnological applications, such as biofertilizer development and the production of value-added compounds, due to its effective nitrogen fixation and potential for metabolic engineering [5,6]. Understanding its metabolic networks is crucial for harnessing its capabilities in synthetic biology and industrial applications. Among its numerous metabolic pathways, the biosynthesis of amino acids, particularly L-lysine (hereafter lysine), has attracted significant interest due to its fundamental and applied implications.

Lysine is an essential amino acid with a vital role in protein synthesis, cellular metabolism, and as a precursor for various secondary metabolites [7]. Its industrial importance is evident in its widespread use in animal feed supplements, pharmaceuticals, and food additives. The microbial production of lysine, primarily through engineered *Corynebacterium glutamicum* and *Escherichia coli* strains, has revolutionized the amino acid industry [8–10]. However, recent advancements in metabolic engineering have opened new possibilities for lysine biosynthesis, utilizing the photosynthetic capabilities of cyanobacteria to decrease carbon input requirements and environmental impact [11,12]. Although photosynthetic organisms hold great promise for lysine production, their metabolic pathways, particularly the functions of crucial enzymes like diaminopimelate decarboxylase (DAPDC), remain underexplored compared with those of conventional microbial hosts.

DAPDC (EC 4.1.1.20) is a pyridoxal 5′-phosphate (PLP)-dependent enzyme that plays a pivotal role in the lysine biosynthesis pathway. Specifically, it is the terminal enzyme to synthesize lysine in the diaminopimelate (DAP) pathway, a highly conserved metabolic route found across bacteria, archaea, and plants [13]. DAPDC catalyzes the decarboxylation of meso-diaminopimelate (meso-DAP) to generate lysine, a reaction that is essential for lysine production. The regulation and activity of this enzyme significantly affect the overall yield of lysine, making it a critical target for optimizing lysine biosynthesis [9]. Beyond its role in amino acid metabolism, DAPDC is also important for bacterial cell wall biosynthesis, as meso-DAP is a necessary precursor for peptidoglycan synthesis, a major structural component of bacterial cell walls [14]. Therefore, understanding the structure-function relationship, catalytic mechanism, and regulation of DAPDC is essential for both basic biology and potential metabolic engineering applications.

While DAPDC has been thoroughly studied in heterotrophic bacteria such as *E. coli* and *Mycobacterium tuberculosis* [15,16], there is limited information on its activity and regulation in cyanobacteria, including *Cyanothece* sp. ATCC 51142. The unique metabolic and regulatory context of *Cyanothece* sp. ATCC 51142, which integrates photosynthesis and nitrogen fixation, might influence DAPDC activity in ways not observed in other microbial systems. Additionally, recent studies suggest that the cyanobacterial lysine biosynthesis pathway is closely linked to nitrogen metabolism and photosynthetic carbon fixation, presenting new regulatory points for optimizing lysine production [17]. Exploring these connections in *Cyanothece* sp. ATCC 51142 could provide valuable insights into the broader metabolic networks of cyanobacteria and guide strategies for enhancing lysine yields through genetic engineering.

Considering the critical role of lysine and DAPDC in microbial metabolism, this present study investigates the DAPDC encoded by the *cce1351* gene in *Cyanothece* sp. ATCC 51142 (hereafter referred to as CsDAPDC). Through an integrated approach involving biochemical characterization, enzyme kinetics, homology modeling, molecular docking, and site-directed mutagenesis, the key residues critical for substrate binding, cofactor interaction, and catalysis were identified. To the best of our knowledge, this present study is the first to report the kinetic and structural characteristics of the DAPDC from cyanobacteria.

## Results

### Sequence analysis and secondary structural prediction of CsDAPDC

Utilizing the ProtParam tool on the Expasy platform, an *in silico* analysis was conducted on the *cce1351* gene. This gene was 1431 base pairs (bp) in length and encoded a protein comprising 476 amino acids. The molecular weight of the predicted CsDAPDC was calculated to be 52.7 kDa, with a theoretical isoelectric point (pI) of 5.46 and an estimated molar extinction coefficient of 54,040 M^-1^·cm^-1^. To predict the secondary structure of the CsDAPDC, the SOMPA online tool was employed, yielding results depicted in Supplementary Figure S1. The secondary structure composition was predicted to consist of 33.19% α-helices, 8.4% β-sheets, 36.97% random coils, and 21.43% extended strands.

### Cloning, expression, and purification of CsDAPDC

**Figure 1:**
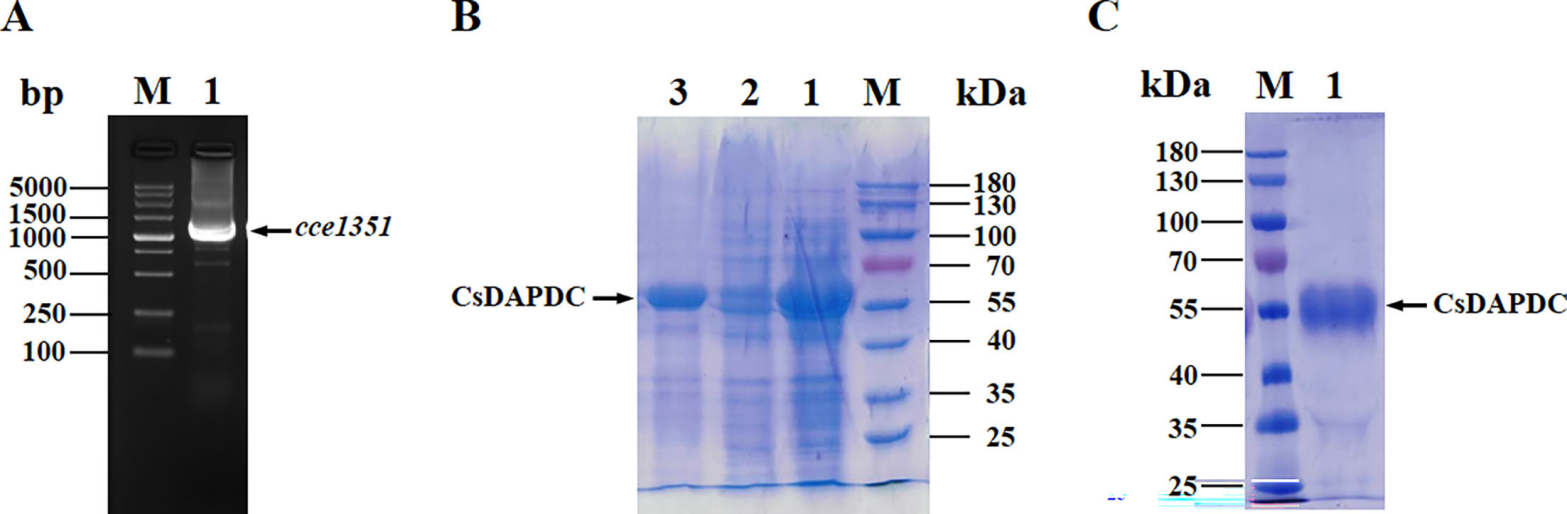
Cloning, expression, and purification of CsDAPDC. (**A**) PCR amplification of cce1351 gene, M: DNA marker, Lane 1: PCR amplification product. (**B**) Expression of recombinant CsDAPDC protein, M: Protein Standards, Lanes 1–3: whole cell lysate, cell pellet and supernatant of plasmid pET28a-cce1351 transformed with *E. coli* BL21 (DE3), respectively. (**C**) The purified CsDAPDC, M: Protein Standards, Lane 1: CsDAPDC. CsDAPDC, *Cyanothece* sp. ATCC 51142 diaminopimelate decarboxylase.

The amplification of the *cce1351* gene encoding CsDAPDC was accomplished by polymerase chain reaction (PCR) utilizing *Cyanothece* sp. ATCC 51142 genomic DNA as a template and commercial primers (Supplementary Table S1). A distinct band of approximately 1500 bp on a 1.5% agarose gel, aligning with the expected size of the *cce1351* gene (1431 bp), was obtained (Figure 1A). The recombinant pET28a-cce1351 plasmid was sequence-verified and subsequently transformed into *E. coli* BL21 (DE3) cells for protein expression. Sodium dodecyl sulfate-polyacrylamide gel electrophoresis (SDS-PAGE) analysis confirmed the successful expression of the CsDAPDC protein with a molecular weight of approximately 55 kDa, which was consistent with the theoretical molecular weight of 52.7 kDa, predominantly in a soluble form (Figure 1B). Nickel-nitrilotriacetic acid (Ni-NTA) affinity chromatography was employed for protein purification, and the CsDAPDC protein was effectively eluted at 100 mM imidazole, yielding a protein preparation with a purity of 90% (Figure 1C and Supplementary Figure S2). The concentration of the purified CsDAPDC protein was determined to be 243 μM, with a yield of approximately 5.82 mg/g (protein/cell mass).

**Figure 2:**
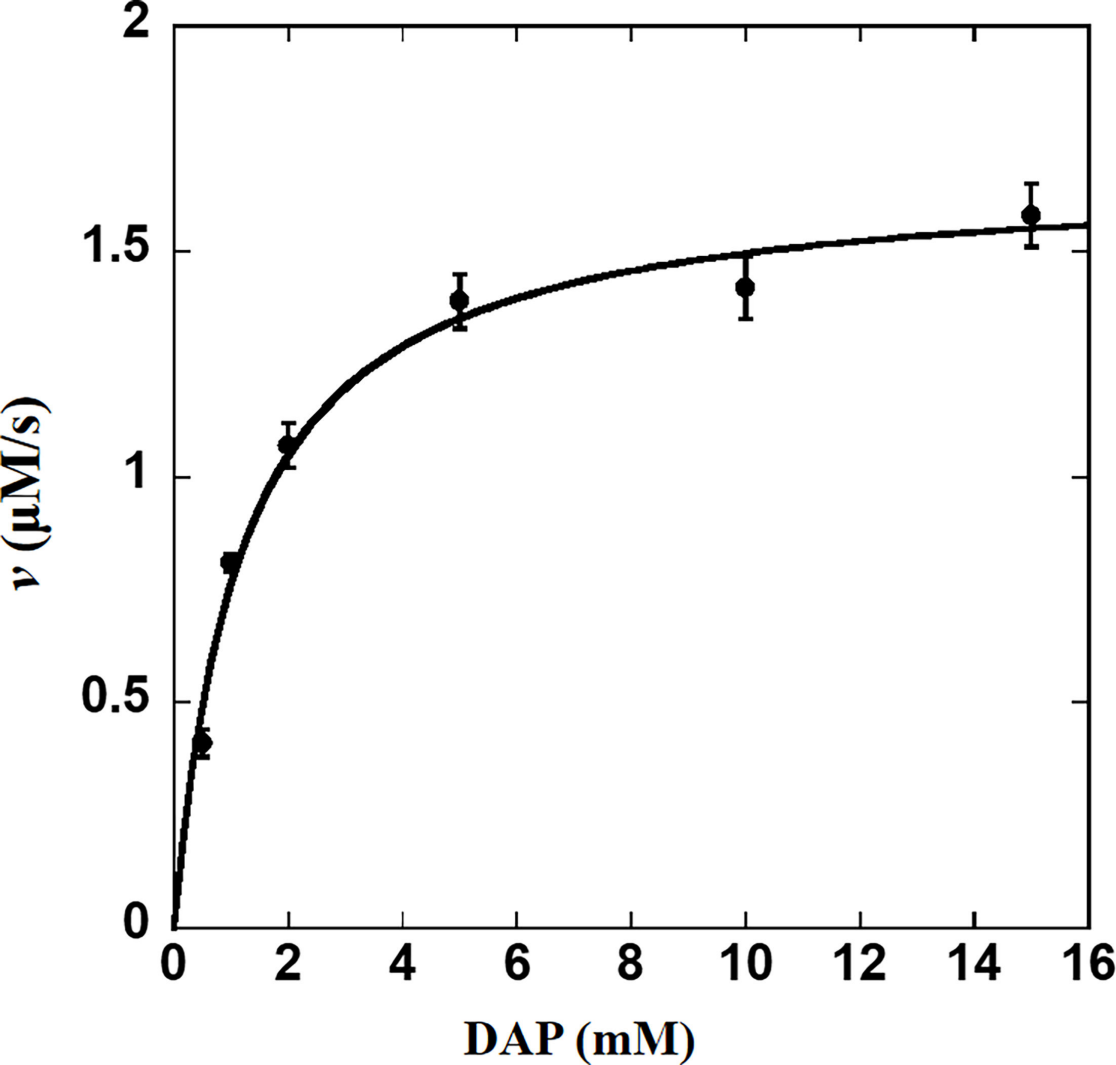
The kinetic analysis of CsDAPDC. The assays were conducted in a reaction mixture comprising 0.2 Mm NADH, 10 mM α-KG, 0.1 mM PLP, 1.4 μM SDH, 1 μM CsDAPDC and a variable concentration of DAP ranging from 0.5 to15 mM in 20 mM Tris-HCl, pH 8.0 buffer. The assays were kept within the linear phase of the initial velocity. All experiments were conducted in triplicate with three independent protein preparations. DAP, diaminopimelate.

### Enzymatic kinetic characterization of CsDAPDC protein

The enzymatic kinetic parameters of CsDAPDC were determined with PLP at a constant concentration of 0.1 mM. The maximal initial velocity (*V*_max_) and Michaelis constant (*K*_M_) were determined to be 1.68 ± 0.06 μM/s and 1.20 ± 0.17 mM, respectively (Figure 2). As a result, the catalytic constant (*k*_cat_) and catalytic efficiency (*k*_cat_/*K*_M_) were calculated to be 1.68 s^-1^ and 1.40 × 10^3^ M^-1^·s^-1^, respectively, with the CsDAPDC concentration of 1 μM.

### Temperature-dependent catalytic activity and stability of CsDAPDC

**Figure 3:**
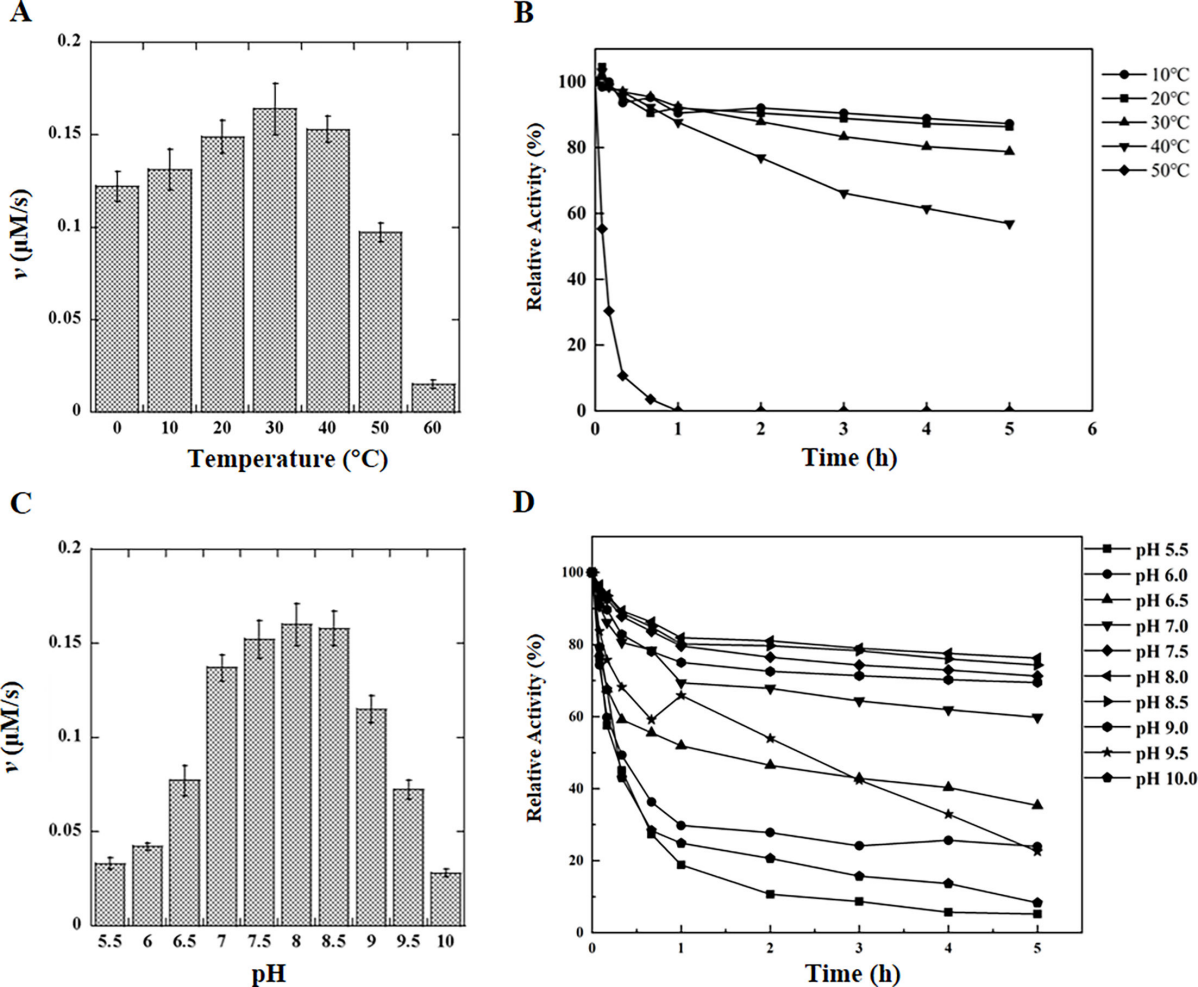
The effects of temperature and pH on the activity and stability of CsDAPDC. (**A**) Effects of temperature on the activity of CsDAPDC. The assays were carried out with 15 mM DAP, 0.1 mM PLP, and 0.1 μM CsDAPDC enzyme in a 20 mM Tris-HCl, pH 8.0 buffer incubated at a temperature ranging from 0 to 60℃ for 10 minutes. (**B**) Effects of temperature on the stability of CsDAPDC. The activity of incubated enzyme (a final concentration of 0.4 μM) was assayed with 15 mM DAP and 0.1 mM PLP in a 20 mM Tris-HCl, pH 8.0 buffer. (**C**) Effects of pH on the activity of CsDAPDC. The assays were carried out with 15 mM DAP, 0.1 mM PLP, and 0.1 μM CsDAPDC enzyme in various pH buffers for 10 minutes. (**D**) Effects of pH on the stability of CsDAPDC. The activity of incubated enzyme (a final concentration of 0.4 μM) was assayed with 15 mM DAP and 0.1 mM PLP in a 20 mM Tris-HCl, pH 8.0 buffer.

The influence of temperature on the catalytic activity of CsDAPDC was examined over a range from 0°C to 60°C. As presented in Figure 3A, the enzyme’s catalytic activity exhibited a temperature-dependent increase, reaching a peak at 30°C. Beyond this temperature, the activity declined progressively with further increases in temperature, showing a near-total loss of activity at 60°C.

The temperature’s impact on the enzyme’s stability was further characterized by monitoring the residual activity across varying temperatures, as depicted in Figure 3B. Notably, at 50°C, CsDAPDC underwent rapid inactivation, with complete denaturation occurring within an hour. In contrast, at lower temperatures, the enzyme’s activity decayed gradually. The rate of activity loss was inversely related to temperature, with lower temperatures correlating with enhanced enzyme stability.

### pH-dependent modulation of activity and stability of CsDAPDC

The influence of pH on the catalytic activity of CsDAPDC was evaluated at a range of pH from 5.5 to 10 (Supplementary Table S2). The enzyme demonstrated catalytic competence within a pH range of 7.0 to 9.0, and its activity was notably diminished at the extremes of pH below 7.0 and above 9.0 (Figure 3C). The pH for peak catalytic activity was identified as 8.0.

The pH environment is a critical determinant of enzyme function, as it directly affects both catalytic efficiency and stability. The residual activity of CsDAPDC under diverse pH conditions is illustrated in Figure 3D. A precipitous decline in the enzyme’s residual activity was observed upon incubation in highly acidic or highly alkaline buffer solutions. Conversely, the enzyme exhibited a more gradual decrease in residual activity in buffer solutions with a pH value of 8.0. Collectively, these observations suggest that CsDAPDC achieves its highest stability in buffer solutions at pH 8.0.

### Modeling and structural evaluation of CsDAPDC

**Figure 4:**
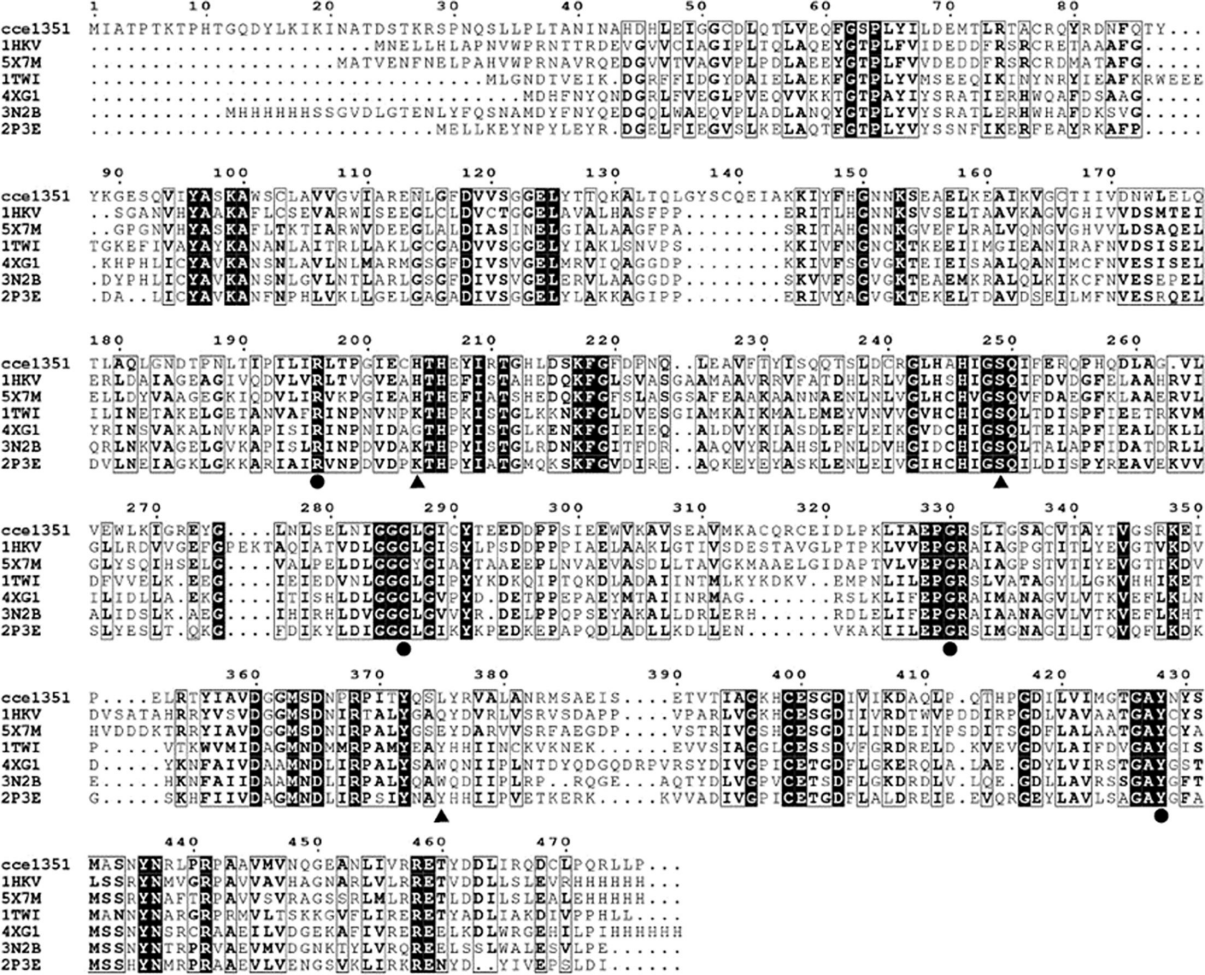
The amino acid sequences alignment of DAPDCs of known structure from different organisms. cce1351: *Cyanothece* sp. ATCC 51142; 1HKV: *Mycobacterium tuberculosis*; 5X7M: *Corynebacterium glutamicum*; 1TWI: *Methanocaldococcus jannaschii*; 4XG1: *Psychromonas ingrahamii*; 3N2B: *Vibrio cholerae*; 2P3E: *Aquifex aeolicus*. The residues bound to DAP are indicated by solid triangle (▲), the residues bound to PLP are represented by solid circle (●).

To analyze the amino acid sequence characteristics of CsDAPDC, we performed a multiple sequence alignment of CsDAPDC and six homologous proteins of known structure from diverse microbial sources. Despite the relatively low sequence identities among these proteins, typically below 40%, certain amino acids were found to be highly conserved, as highlighted by the shaded boxes (Figure 4). Notably, some key residues near PLP and DAP in other DAPDCs were also conserved in CsDAPDC, marked with solid circles and triangles, respectively (Figure 4).

**Figure 5:**
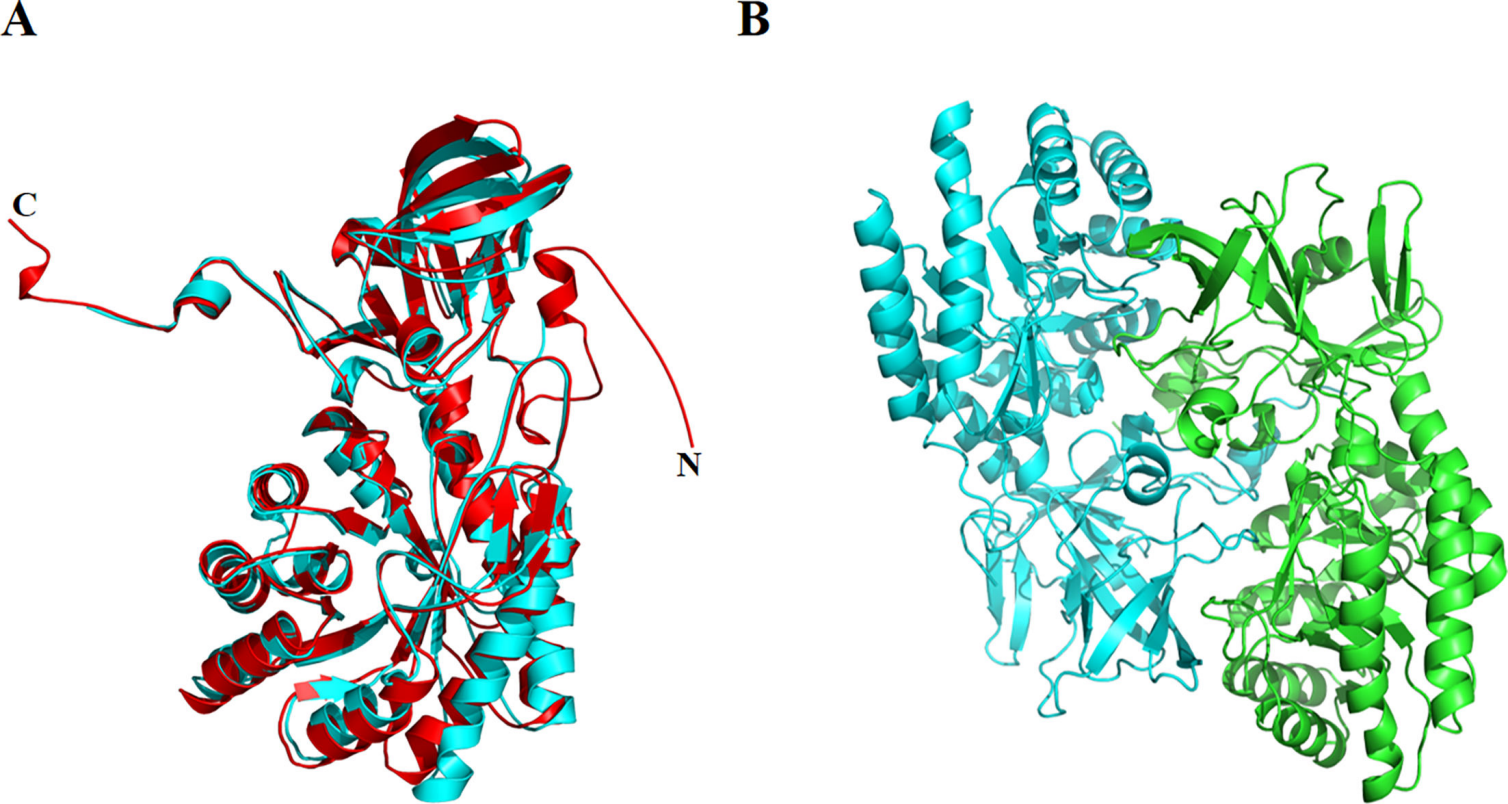
The predicted three-dimensional structure of CsDAPDC. (**A**) The aligned structures of CsDAPDC predicted by SWISS-MODEL (cyan) and AlphaFold2 (red). (**B**) The dimer structure of CsDAPDC predicted by SWISS-MODEL. The two chains are shown in cyan and green, respectively.

The model structure of CsDAPDC predicted by SWISS-MODEL and the structure computed from AlphaFold2 were aligned in PyMol 1.3 .X, revealing a high degree of structural similarity with a root-mean-square deviation (RMSD) of 0.98 Å under default settings (Figure 5A). This value reflected the highly similar overall folding and spatial arrangements of the two structures, despite minor differences in certain local regions (Figure 5A). Meanwhile, a model dimer structure of CsDAPDC was also generated by SWISS-MODEL (Figure 5B). Both modeled structures closely resembled the crystal structure of DAPDC from *M. tuberculosis* (PDB ID: 1HKV). The quality of the model structure of CsDAPDC was initially assessed using Global Model Quality Estimation (GMQE) and Qualitative Model Energy Analysis (QMEAN) scores obtained from the SWISS-MODEL assessment platform. GMQE scores range from 0 to 1, with higher values indicating greater expected model accuracy, whereas QMEAN scores range from −4 to 0, with values closer to 0 signifying greater congruence between the model and the template. For the CsDAPDC model structure, the GMQE score of 0.71 and the QMEAN score of −1.50 suggested strong structural internal consistency (Supplementary Figure S3). To further evaluate the model’s reliability, a Ramachandran plot was generated using PROCHECK to analyze the dihedral angles of the protein backbone and categorize amino acids into favorable and unfavorable regions [18]. The results (Supplementary Figure S4 and Table S3) revealed that 99.6% of amino acid residues resided in favorable regions, while only a minor fraction was found in disallowed regions. This distribution underscores the modeled structure’s high reliability.

### Active sites and molecular docking analysis of CsDAPDC

**Figure 6:**
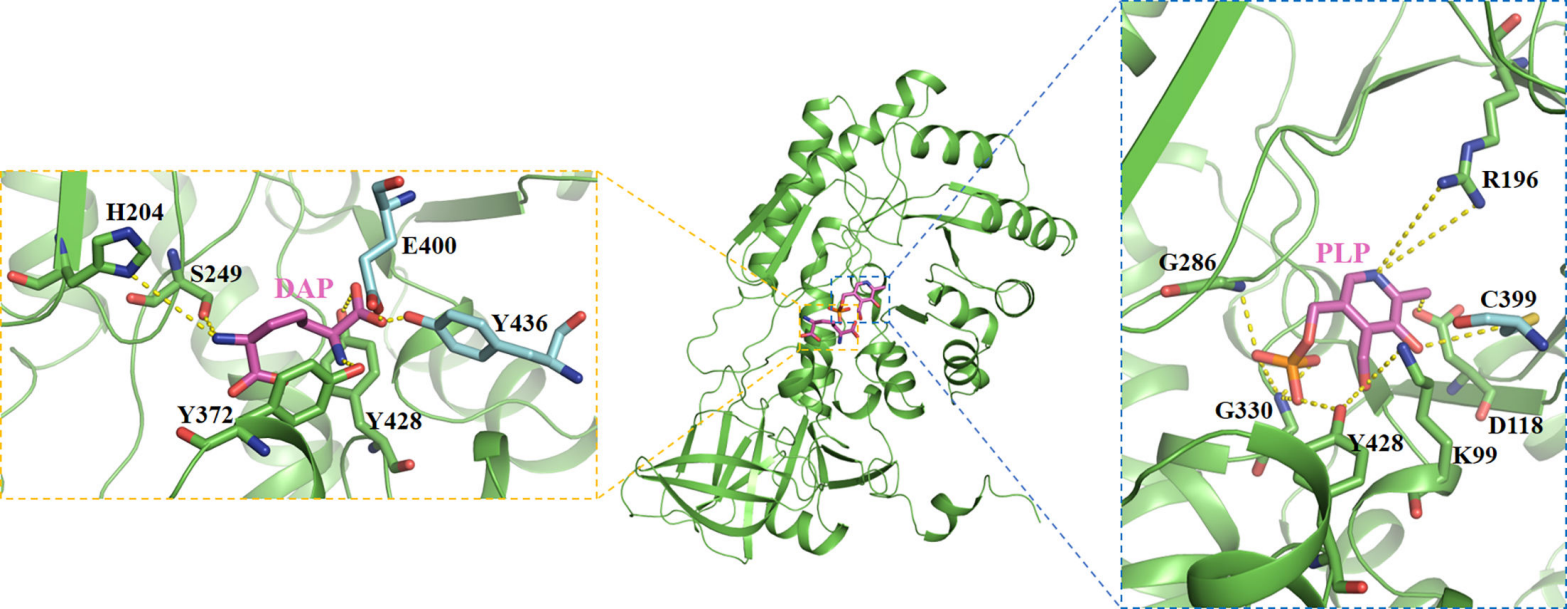
The molecular docking of CsDAPDC with DAP (left) and PLP (right). The residues involved in interacting with DAP/PLP were shown as sticks. The carbons in residues from the other monomer were colored cyan. DAP, diaminopimelate.

**Figure 7:**
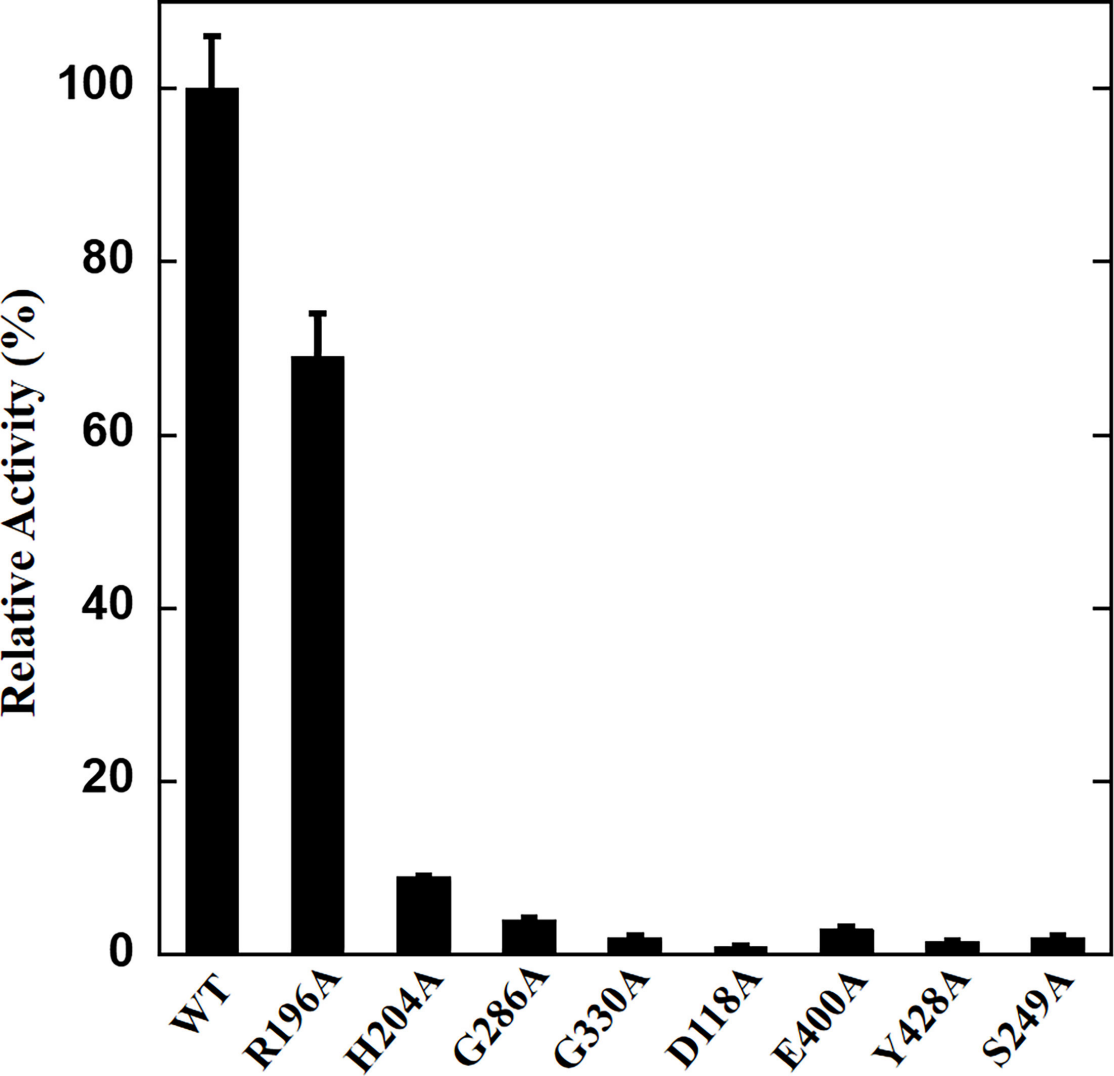
Site-directed mutagenesis of CsDAPDC. The relative activities of mutants were determined and compared with that of wildtype CsDAPDC. WT, wildtype.

Alignment of amino acid sequences demonstrated that CsDAPDC possesses a highly conserved ‘pocket’ structure, shared with other DAPDCs (Figure 4), which serves as an active site. Consequently, the binding regions of CsDAPDC for DAP and PLP were preliminarily identified. These regions were designated as docking sites for subsequent molecular docking simulations. Twenty docking conformations were generated, representing predicted interactions between CsDAPDC and DAP/PLP. The docking conformation exhibiting the lowest free energy and most stable bonding interactions was selected as the final result (Figure 6 and Supplementary Table S4). Molecular docking results revealed that the residues of Ser249, His204, Tyr372, and Tyr428 interacted with the substrate of DAP, whereas residues of Asp118, Gly286, Gly330, Lys99, and Tyr428 established strong interactions with the cofactor of PLP (Figure 6). The distances between the residues and DAP/PLP were measured to range from 1.76 Å to 3.92 Å. At the same time, the residues of Cys399, Glu400, and Tyr436 from the other monomer participated in stabilizing the DAP and PLP (Figure 6). To confirm the interactions between CsDAPDC and DAP/PLP, site-directed mutagenesis was performed on certain conserved residues and the mutant proteins were purified (Supplementary Table S1 and Figure S5). The enzymatic activities analysis of these mutants revealed that, with the exception of the R196A mutant, the other seven mutants almost completely lost their ability to catalyze DAP conversion to lysine. In contrast, the R196A mutant retained approximately 69% of the wildtype activity (Figure 7).

## Discussion

In this present study, the DAPDC from *Cyanothe* sp. ATCC 51142 (CsDAPDC), a key enzyme in the lysine biosynthesis pathway, was successfully cloned, expressed, and purified (Figure 1). Additionally, a preliminary biochemical and structural characterization of CsDAPDC was conducted, providing knowledge of kinetic parameters, enzyme structure, and essential residues of cyanobacterial DAPDC and its potential role in lysine production.

Enzymatic kinetic analysis of CsDAPDC revealed a substrate binding affinity (*K*_M_) of 1.20 mM and a *k*_cat_ of 1.68 s^-1^. These values were comparable to those of DAPDCs characterized in heterotrophic bacteria. For instance, the *K*_M_ values for DAP with DAPDCs from *E. coli* and *M. tuberculosis* were determined to be 0.97 mM and 1.6 mM, respectively [15]. Additionally, the *k*_cat_ of DAPDC from *M. tuberculosis* was found to be 1.8 s^-1^ at pH 7.8 and 25℃ [16], while that of DAPDC from *Helicobacter pylori* was 15.65 s^-1^ at pH 8.0 and 30℃ [19]. These findings might suggest that CsDAPDC has comparable substrate recognition and binding mechanisms to other DAPDCs.

The peak activity of CsDAPDC was observed at pH 8.0 and 30°C (Figure 3). The enzyme’s stability at moderate temperatures and alkaline pH renders it amenable for biotechnological processes, particularly in the context of lysine production [11,12]. The enzyme exhibited significant activity across a broad pH spectrum (7.0–9.0), with a peak at pH 8.0 (Figure 3), which was in harmony with those reported for homologous enzymes in other species such as *E. coli*, *M. tuberculosis*, *Bacillus subtilis*, and *Bacillus anthracis* [15,20]. Meanwhile, the temperature of highest activity was 30°C for CsDAPDC, which was also observed in counterparts from the above four bacteria [21].

The glycine-rich motif (Gly284, Gly285, and Gly286), the EPGR motif (Glu328, Pro329, Gly330, and Arg331), and the GAY motif (Gly426, Ala427, and Tyr428) were conserved in the PLP binding pocket, which interacted with the phosphate moiety of the PLP cofactor (Figures 4 and 6). The Arg196, Lys99, and Asp118 residues contributed to stabilize the pyridoxal ring of the PLP cofactor through hydrogen bonds (Figure 6). Notably, the Cys399 residue from the other monomer also interacted with the pyridoxal ring of PLP (Figures 5B and 6). Meanwhile, the results from AutoDock suggested that Ser249, His204, and Tyr372 residues were involved in directly interacting with the two amino groups of the DAP substrate, while the Tyr428 residue and the Glu400 and Tyr436 residues from the other monomer participated in binding the carboxyl group of DAP (Figures 5B and 6). This observation indicated that the protein dimerization was important for the activity of CsDAPDC [15,22]. In fact, the active site of most DAPDCs was at the dimer interface, with the residues from both monomers forming the catalytic center [15,22–24]. Site-directed mutagenesis of certain conserved residues confirmed the functional importance of these residues, as most mutants exhibited a nearly complete loss of enzymatic activity (Figure 7).

In summary, this present study provides the first preliminary characterization of DAPDC from a cyanobacterial species. The insights gained from the kinetic, structural, and mutagenesis analyses of CsDAPDC advance our understanding of lysine biosynthesis in *Cyanothece* species.

## Methods

### Physicochemical property analysis and secondary structure prediction of CsDAPDC

The online tool ProtParam (https://web.expasy.org/protparam/, accessed on December 30, 2024) was used to predict the amino acid sequences, the theoretical pI, the molar extinction coefficient, and other physicochemical properties of CsDAPDC. Subsequently, the amino acid sequences of CsDAPDC were uploaded to the online tool SOPMA ( https://npsa.lyon.inserm.fr/cgi-bin/npsa_automat.pl?page=/NPSA/npsa_sopma.html, accessed on December 30, 2024) to predict the secondary structure of CsDAPDC.

### Plasmid construction, protein expression, and purification

The *cce1351* gene was amplified from *Cyanothece* sp. ATCC 51142 genomic DNA using commercial primers (Supplementary Table S1), and the amplicon was subjected to double-digestion with the restriction endonucleases of *NdeI* and *XhoI*. The *cce1351* gene fragment was ligated into the pET-28a vector, which was cut with the same two endonucleases, to construct a recombinant pET28a-cce1351 plasmid.

The recombinant *E. coli* BL21 (DE3) strain harboring the pET28a-cce1351 plasmid was used to express the CsDAPDC protein. The strain was cultured overnight and subsequently diluted 1:100 into Luria–Bertani (LB) medium supplemented with kanamycin at a concentration of 50 μg/ml to ensure plasmid selection. Upon reaching an optical density at 600 nm (OD_600_) of approximately 0.6, the culture was induced with isopropyl β-D-thiogalactoside at a final concentration of 0.2 mM. Induction was performed at a reduced temperature of 16°C for a duration of 24 hours to optimize protein folding and solubility.

Post induction, the bacterial cells were harvested by centrifugation, resuspended in a buffer comprising 20 mM Tris-HCl and 300 mM NaCl at a pH of 7.5, and subjected to ultrasonication to achieve cell lysis. The resulting lysate was separated into supernatant and pellet fractions, which were analyzed via SDS-PAGE to confirm the expression of the recombinant protein. For purification, the clarified lysate was centrifuged to remove cellular debris, filtered through a 0.45 μm membrane, and then applied to an Ni-NTA resin that had been preconditioned to bind His-tagged proteins. The bound protein was eluted using a stepwise imidazole gradient ranging from 20 to 200 mM, and the elution fractions were analyzed by SDS-PAGE to assess purity. Fractions exhibiting high purity were pooled, dialyzed against the 20 mM Tris-HCl and 300 mM NaCl at a pH of 7.5 buffer to remove imidazole, and concentrated using polyethylene glycol 20,000 to the appropriate concentration. The final purified protein was quantified via UV spectrophotometry at 280 nm with a molar extinction coefficient (ε) of 54,040 M^-1^·cm^-1^.

### Enzymatic activity determination of CsDAPDC

The enzymatic activity of the recombinant CsDAPDC was determined using a coupled spectrophotometric assay, utilizing saccharopine dehydrogenase (SDH) as the auxiliary enzyme at room temperature [21]. Briefly, the assay was conducted in a reaction mixture comprising 0.2 mM reduced nicotinamide adenine dinucleotide (NADH), 10 mM α-ketoglutarate (α-KG), 0.1 mM PLP, 1.4 μM SDH, and a variable concentration of DAP ranging from 0.5 to 15 mM in 20 mM Tris-HCl, pH 8.0 buffer. Subsequently, 1 μM CsDAPDC was introduced into the reaction mixture to trigger the enzymatic reaction. The rate of NADH consumption was monitored by measuring the absorbance at 340 nm using a microplate reader. The specific activity of CsDAPDC was then quantified based on the initial rate of absorbance decrease at 340 nm and correlated with the concentration of DAP utilized in the assay. All experiments were conducted in triplicate with three independent protein preparations to ensure the reproducibility and statistical significance of the enzymatic activity measurements.

### Impact of temperature on the activity and stability of CsDAPDC

A two-phase experimental approach was meticulously executed to investigate the temperature-dependent effects on the catalytic efficiency and structural integrity of CsDAPDC. Initially, a reaction mixture comprising 15 mM DAP, 0.1 mM PLP, and 0.1 μM CsDAPDC enzyme in a 20 mM Tris-HCl buffer solution at pH 8.0, with a total volume of 500 μl was incubated at a temperature ranging from 0 to 60℃ for 10 minutes, followed by a thermal denaturation step at 99.5℃ for 5 minutes to inactivate the enzyme. Subsequent centrifugation at 12,000 rpm was employed to isolate the supernatant, which contained the enzyme’s soluble products. In the subsequent phase, a distinct reaction system was formulated, consisting of 0.2 mM NADH, 10 mM α-KG, 1.4 μM SDH, and 20 μl of the aforementioned supernatant, all within a 20 mM Tris-HCl buffer solution at pH 8.0, with a total volume of 200 μl. The enzymatic activity was inferred from the absorbance changes at 340 nm, which was monitored spectrophotometrically. Each trial was performed in triplicate.

The thermal stability of the recombinant CsDAPDC was evaluated. The enzyme, at a final concentration of 0.4 μM, was incubated within the temperature range of 10 to 50℃ in a 20 mM Tris-HCl buffer solution at pH 8.0 for defined periods, varying from 5 minutes to 5 hours. The residual enzymatic activity was then quantified using the aforementioned continuous spectrophotometric protocols. All experiments were performed in triplicate.

### pH-dependent catalytic activity and stability of CsDAPDC

The effect of pH on the catalytic activity and stability of CsDAPDC was investigated using a two-step approach similar to the previous section. The initial reaction mixture (500 μl) containing 15 mM DAP, 0.1 mM PLP, and 0.1 μM CsDAPDC in various pH buffers as detailed in Supplementary Table S2 was incubated at room temperature for 10 minutes, followed by thermal inactivation of the enzyme at 99.5℃ for 5 minutes and centrifugation at 12,000 rpm to isolate the supernatant. The residual enzymatic activity was then quantified using the aforementioned protocols. All experiments were performed in triplicate.

To evaluate the pH-dependent stability of CsDAPDC, the protein was incubated in a spectrum of pH buffers as listed in Supplementary Table S2 for a specific time, varying from 5 minutes to 5 hours. The residual enzymatic activity was then quantified using the aforementioned protocols. All experiments were performed in triplicate.

### Homology modeling and molecular docking analysis of CsDAPDC

A homology model of CsDAPDC was constructed employing SWISS-MODEL (https://swissmodel.expasy.org/, accessed on September 25, 2024) utilizing the crystal structure of DAPDC from *M. tuberculosis* (PDB ID: 1HKV) as the template [25]. At the same time, the structure of CsDAPDC was calculated by AlphaFold2 [26]. The model structure of CsDAPDC from SWISS-MODEL was evaluated by the SWISS-MODEL platform [27]. Subsequently, molecular docking simulations were conducted to elucidate the binding interactions between CsDAPDC and its ligands, DAP and PLP, using AutoDock Tools 1.5.7 [28]. The Lamarckian genetic algorithm was implemented within a semi-flexible docking framework to scrutinize the interactions between CsDAPDC and ligands. The homology-modeled structure of CsDAPDC was exported in PDB format, while the molecular structure files for DAP and PLP were sourced from the PubChem database [29] and subsequently converted into PDBQT format, which is compatible with AutoDock. Prior to docking, the PDB file of CsDAPDC was preprocessed using AutoDock software. This preprocessing involved the addition of hydrogen atoms to account for the hydrogen bonding potential and the balancing of charges to reflect the electrostatic properties of the protein. The docking results were converted into PDB format using Open Babel GUI and visualized in PyMOL 1.3 .X.

### Site-directed mutagenesis of CsDAPDC key residues

Key residues identified through docking and sequence alignment were mutated to alanine using fast mutagenesis technology with pET28a-cce1351 wildtype plasmid as template and commercial primers (Supplementary Table S1). The mutated plasmid was digested in a 30 μl reaction system consisting of 26 μl gel-purified product, 3 μl 10× QuickCut Buffer, and 1 μl QuickCut Dpn I enzyme. The reaction was incubated at 37℃ for 2 hours. The digested pET28a-cce1351 mutant plasmid was subsequently transformed into *E. coli* DH5α competent cells and plated onto LB agar supplemented with 50 μg/ml kanamycin. All the mutated plasmids were verified by sequencing. The recombinant mutant proteins were expressed and purified employing the same protocols as those for wildtype protein. The catalytic activities of the mutants were determined according to the method described for the wildtype protein.

## Supplementary material

Online supplementary material 1

## Data Availability

The data generated or analyzed during this study are included in the main article and its supplementary files.

## Open Access

This article has been published open access under our Subscribe to Open programme, made possible through the support of our subscribing institutions, learn more here: https://portlandpress.com/pages/open_access_options_and_prices#conditional

## Abbreviations

CsDAPDC: *Cyanothece* sp. ATCC 51142
DAP: diaminopimelate
DAPDC: diaminopimelate decarboxylase
GMQE: Global Model Quality Estimation
*K*_M_: Michaelis constant
NADH: nicotinamide adenine dinucleotide
Ni-NTA: nickel-nitrilotriacetic acid
PLP: pyridoxal 5′-phosphate
QMEAN: Qualitative Model Energy Analysis
SDH: saccharopine dehydrogenase
SDS-PAGE: sodium dodecyl sulfate-polyacrylamide gel electrophoresis
bp: base pair
*k*_cat_: catalytic constant
meso-DAP: meso-diaminopimelate
pI: isoelectric point
α-KG: α-ketoglutarate
