# Supplementary material for: Kinetic and homology model analysis of diaminopimelate decarboxylase from Cyanothece sp. ATCC 51142: unveiling a key enzyme in lysine biosynthesis
Source: Biosci Rep. 2025 Sep 18;45(9):505–16. doi: 10.1042/BSR20253430 (PMC12599289; doi:10.1042/BSR20253430)
Supplement: Online supplementary material 1 [file bsr-45-09-BSR20253430-s001.docx]

**Kinetic and Homology Model Analysis of Diaminopimelate Decarboxylase from *Cyanothece* sp. ATCC 51142: Unveiling A Key Enzyme in Lysine Biosynthesis**

Zhi-Min Li^1^, Suhang Chen^2^, Weikang Luo^2^, Fang Wang^3^, Siqi Wang^2^, Liyang Huang^2^, Xinyue Xiong^2^, Congcong Xie^4^, Zhimin Li^1, 2, *^

^1^College of Chemistry and Materials, Jiangxi Agricultural University, Nanchang, Jiangxi 330045, China; ^2^College of Bioscience and Bioengineering, Jiangxi Provincial Key Laboratory for Postharvest Storage and Preservation of Fruits and Vegetables, Jiangxi Agricultural University, Nanchang, Jiangxi 330045, China; ^3^Jiangxi Provincial Psychiatric Hospital, Nanchang, Jiangxi 330000, China; ^4^School of Biological and Environmental Engineering, Jingdezhen University, Jingdezhen, Jiangxi 333000, China.

*Correspondence: Zhimin Li ([zhiminli@jxau.edu.cn](mailto:zhiminli@jxau.edu.cn))

**Legends**

**Table S1.** Sequences of primers used in this study

**Table S2.** Different pH buffers used in this study

**Table S3.** Ramachandran plot statistics of CsDAPDC model structure computed with PROCHECK program

**Table S4.** Cluster analysis of docking conformations

**Figure S1.** The predicted secondary structure of CsDAPDC. Blue represents alpha helix, green represents beta turn, yellow represents random coil, and purple represents extended strand.

**Figure S2.** The purification of CsDAPDC wild type protein. M: mixed broad molecular weight protein standards; Lane 1: Cell lysate after induction of expression; Lane 2: Supernatant after cell disruption and centrifugation; Lane 3: Cell fragmentation solution was centrifuged and pelleted and resuspended; Lane 4: Supernatant flow through Ni-NTA flow through; Lanes 5~9: 20 mM, 40 mM, 60 mM, 80 mM and 100 mM imidazole elution, respectively; Lanes 10-13: all 100 mM imidazole elution.

**Figure S3.** Evaluation of model structure quality of CsDAPDC by selected SWISS-MODEL assessment tools.

**Figure S4.** Ramachandran plot of CsDAPDC predicted structure.

**Figure S5.** The construction and purification of CsDAPDC mutants. **A**: PCR amplification product of pET28a-cce1351 site-directed mutation. **B**: PCR amplification product of pET28a-cce1351 site-directed mutant colony. **C**: purification of recombinant Y428A mutant protein. M: mixed broad molecular weight protein standards; Lane 1: cell lysate after induction of expression; Lane 2: supernatant after cell disruption and centrifugation; Lane 3: cell fragmentation solution was centrifuged and pelleted and resuspended; Lane 4: supernatant flow through Ni-NTA flow through; Lanes 5~9: 20 mM, 40 mM, 60 mM, 80 mM and 100 mM imidazole elution, respectively; Lanes 10-14: all 100 mM imidazole elution.

**Table S1**. Sequences of primers used in this study

| **Proteins** | **Primers** | **Primer Sequences** |
| --- | --- | --- |
| Wild type | cce1351-F | GC***CATATG***ATAGCGACACCAACCAAAACGCC |
|  | cce1351-R | C***CTCGAG***TTAGGGTAGAAGCCTTTGGGGTAAACA |
| H204A | H204A-F | CCCAGGTATCGAATGT***GC***TACCCACGAA |
|  | H204A-R | ***CG***ACATTCGATACCTGGGGTCAGTCTGA |
| S249A | S249A-F | GATTACACGCCCACATTGGT***G***CCCAAATCTTC |
|  | S249A-R | ***C***ACCAATGTGGGCGTGTAATCCTCGACAAT |
| E400A | E400A-F | GCTGGAAAACACTGTG***CG***TCTGGGGATATTG |
|  | E400A-R | ***CG*** CACAGTGTTTTCCAGCAATGGTGACGG |
| D118A | D118A-F | GGGAAAACTTAGGCTTTG***C***TGTCGTGTCAG |
|  | D118A-R | ***G***CAAAGCCTAAGTTTTCCCTGGCGATGAC |
| R196A | R196A-F | CCATTCCCATCCTCATC***GCG***CTGACCCCAGG |
|  | R196A-R | ***CGC***GATGAGGATGGGAATGGTCAGGTTCGG |
| G286A | G286A-F | AACTCAACATCGGCGGAG***C***ACTAGGAATTTG |
|  | G286A-R | ***G***CTCCGCCGATGTTGAGTTCTGATAAATTGAGTC |
| G330A | G330A-F | AAATTGATAGCAGAACCAG***C***GCGATCGCTCA |
|  | G330A-R | ***G***CTGGTTCTGCTATCAATTTGGGGAGGTCT |
| Y428A | Y428A-F | CATGGGGACAGGAGCC***GC***CAACTATAGT |
|  | Y428A-R | ***GC***GGCTCCTGTCCCCATGATTACTAGAAT |

Note: endonuclease sites are marked in purple italic bold. Mutation sites are marked in red italic bold.

**Table S2**. Different pH buffers used in this study

| **pH** | **buffers** | **Full Name** | **Concentration (mM)** |
| --- | --- | --- | --- |
| 5.5-6.5 | Bis-Tris | Bis(2-hydroxyethyl)amino-tris(hydroxymethyl)methane | 200 |
| 7.0-7.5 | HEPES | N-2-hydroxyethylpiperazine-N-2-ethane sulfonic acid | 200 |
| 8.0-8.5 | Tris-HCl | Tris(hydroxymethyl)aminomethane hydrochloride | 200 |
| 9.0-9.5 | CHES | N-cyclohexyltaurine | 200 |
| 10 | CAPS | 3-(cyclohexylamino)-1-propanesulfonic acid | 200 |

**Table S3**. Ramachandran plot statistics of CsDAPDC model structure computed with PROCHECK program

| Residue regions | Proportion (%) |
| --- | --- |
| residues in most favored regions | 88.6 |
| residues in additional allowed regions | 10.4 |
| residues in generously allowed regions | 0.6 |
| residues in disallowed regions | 0.4 |

**Table S4**. Cluster analysis of docking conformations

| **Rank** | **Sub-Rank** | **Run** | **Binding Energy (kcal/mol)** | **Cluster RMSD** | **Reference RMSD** |
| --- | --- | --- | --- | --- | --- |
| 1 | 1 | 18 | -5.49 | 0.00 | 235.09 |
| 1 | 2 | 16 | -5.36 | 1.09 | 235.03 |
| 1 | 3 | 9 | -5.31 | 0.52 | 235.02 |
| 1 | 4 | 8 | -4.98 | 0.71 | 234.83 |
| 1 | 5 | 7 | -4.95 | 0.98 | 235.33 |
| 1 | 6 | 20 | -4.92 | 0.75 | 234.98 |
| 1 | 7 | 11 | -4.89 | 1.24 | 235.03 |
| 1 | 8 | 5 | -4.66 | 1.21 | 235.59 |
| 1 | 9 | 15 | -3.74 | 1.93 | 235.25 |
| 1 | 10 | 1 | -3.61 | 1.71 | 235.43 |
| 1 | 11 | 13 | -3.58 | 1.69 | 235.41 |
| 1 | 12 | 10 | -3.50 | 1.46 | 235.39 |
| 1 | 13 | 12 | -2.58 | 1.97 | 235.54 |
| 1 | 14 | 17 | -2.39 | 1.88 | 235.39 |
| 1 | 15 | 2 | -2.22 | 1.88 | 235.79 |
| 2 | 1 | 4 | -4.58 | 0 | 235.30 |
| 2 | 2 | 14 | -3.05 | 1.23 | 235.41 |
| 3 | 1 | 3 | -3.08 | 0 | 235.11 |
| 3 | 2 | 19 | -2.00 | 1.97 | 235.33 |
| 4 | 1 | 6 | -2.67 | 0 | 235.76 |


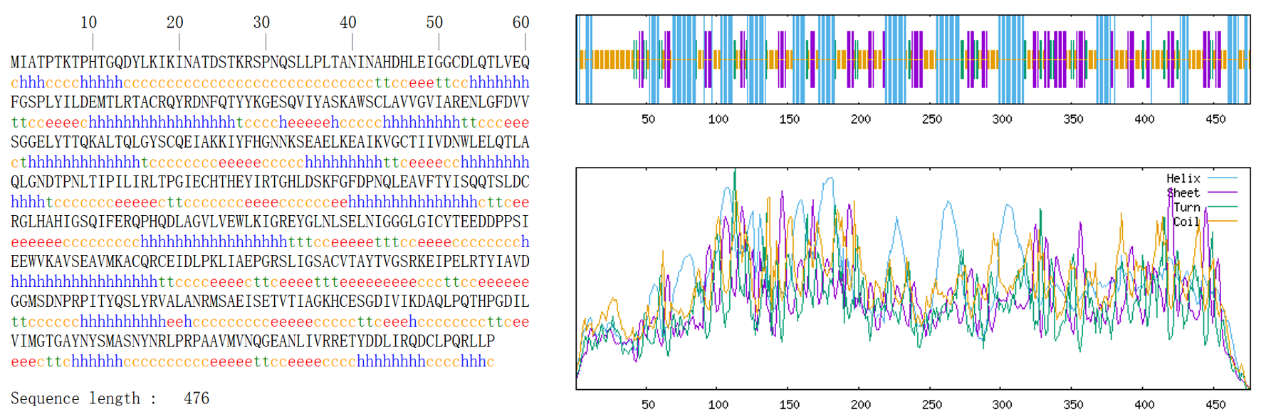


**Figure S1**. The predicted secondary structure of CsDAPDC. Blue represents alpha helix, green represents beta turn, yellow represents random coil, and purple represents extended strand.


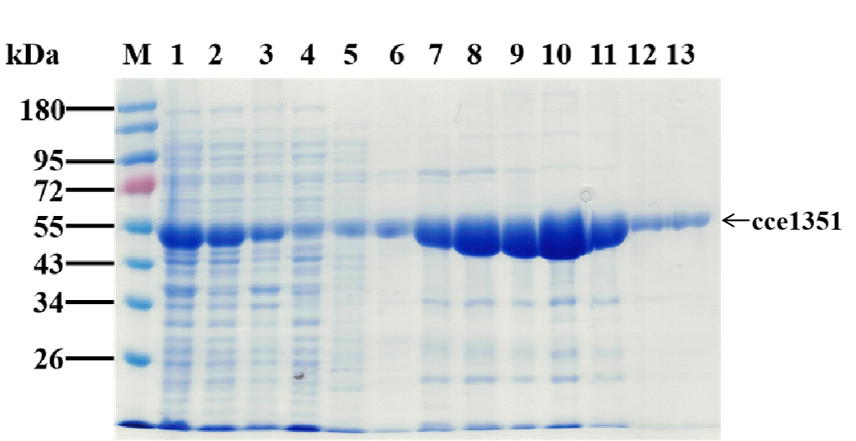


**Figure S2**. The purification of CsDAPDC wild type protein. M: mixed broad molecular weight protein standards; Lane 1: Cell lysate after induction of expression; Lane 2: Supernatant after cell disruption and centrifugation; Lane 3: Cell fragmentation solution was centrifuged and pelleted and resuspended; Lane 4: Supernatant flow through Ni-NTA flow through; Lanes 5~9: 20 mM, 40 mM, 60 mM, 80 mM and 100 mM imidazole elution, respectively; Lanes 10-13: all 100 mM imidazole elution.


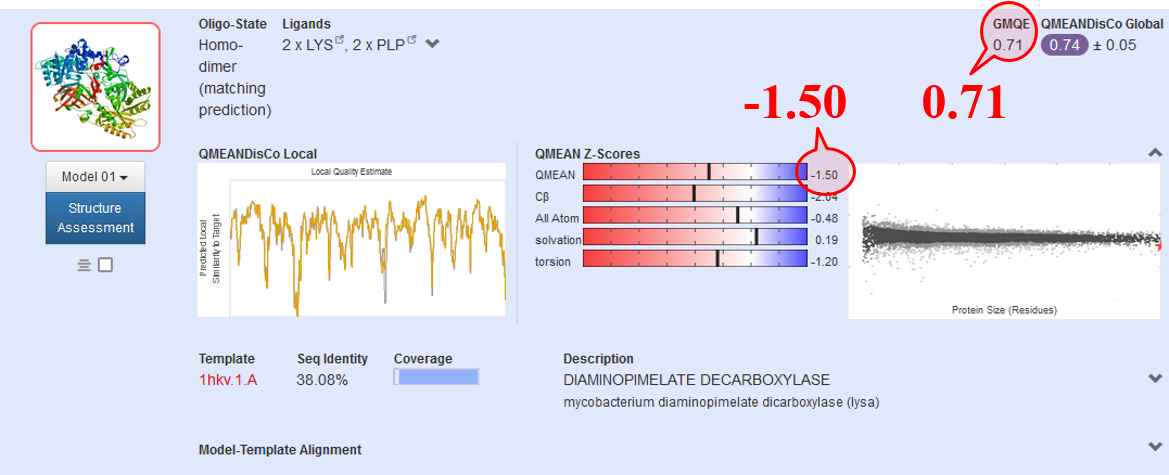


**File S3**. Evaluation of model structure quality of CsDAPDC by selected SWISS-MODEL assessment tools.


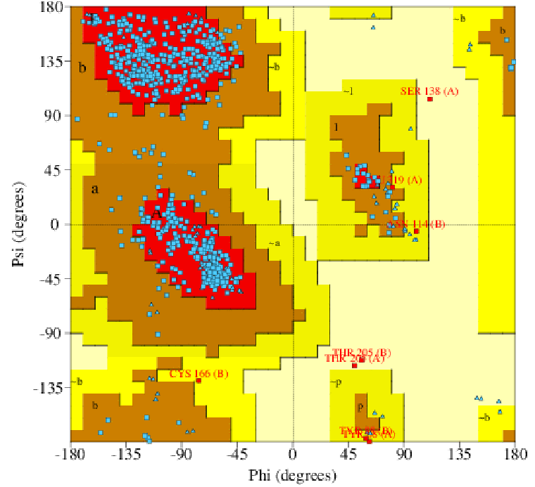


**File S4**. Ramachandran plot of CsDAPDC predicted structure.


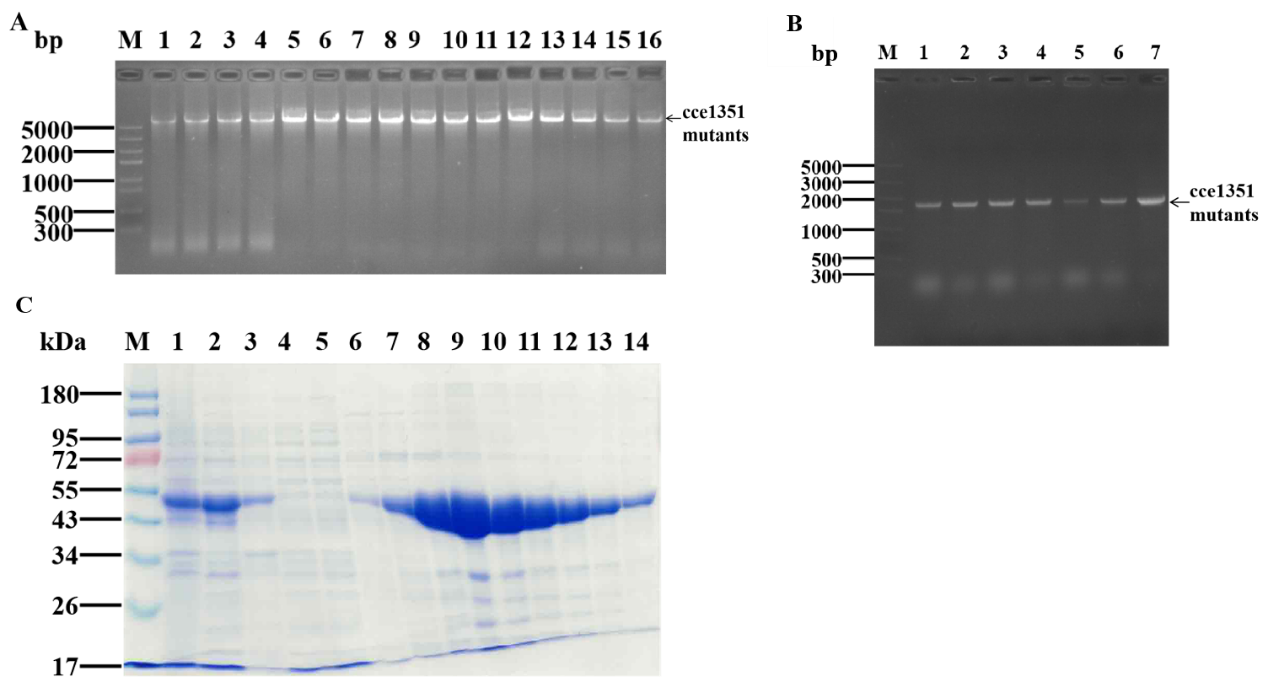


**Figure S5**. The construction and purification of CsDAPDC mutants. **A**: PCR amplification product of pET28a-cce1351 site-directed mutation. **B**: PCR amplification product of pET28a-cce1351 site-directed mutant colony. **C**: purification of recombinant Y428A mutant protein. M: mixed broad molecular weight protein standards; Lane 1: cell lysate after induction of expression; Lane 2: supernatant after cell disruption and centrifugation; Lane 3: cell fragmentation solution was centrifuged and pelleted and resuspended; Lane 4 : supernatant flow through Ni-NTA flow through; Lanes 5~9: 20 mM, 40 mM, 60 mM, 80 mM and 100 mM imidazole elution, respectively; Lanes 10-14: all 100 mM imidazole elution.
